# Reply to: “The data do not support the existence of an ‘Old Boy network’ in science. Some critical comments on a study by Massen et al.”

**DOI:** 10.1038/s41598-020-69108-6

**Published:** 2020-08-14

**Authors:** Jorg J. M. Massen, Lisa Bauer, Benjamin Spurny, Thomas Bugnyar, Mariska E. Kret

**Affiliations:** 1 Animal Ecology Group, Utrecht University, Utrecht, The Netherlands; 2 Department of Behavioural and Cognitive Biology, University of Vienna, Vienna, Austria; 3 Department of Psychiatry and Psychotherapy, Medical University of Vienna, Vienna, Austria; 4 Cognitive Psychology Unit, Leiden University, Leiden, The Netherlands

**Keywords:** Evolution, Psychology

**replying to**: F. Van  de Velde and B. Heller; *Scientific Reports* 10.1038/s41598-020-69278-3 (2020).

## Introduction

We commend Van de Velde and Heller for their goal of keeping high scientific standards, and underline the importance of replication. We too are vested in such standards and are genuinely interested in the results of alternative statistical analyses. That said, we feel that the reported results do not harm the conclusions from our original paper, namely, that male scientists are very likely to follow up on requests to share their science with other males, and that such helping behavior was less frequently observed between any other sex combination of requester and participant^1^. Whereas the alternative analyses presented by Van de Velde and Heller may cast some uncertainty on the found effect, they did yield comparable results, and importantly showed a similar trend (*P* = 0.061) for the reported interaction. We hope that our disagreement about the specific analyses will not distract us from the core problem: structural gender biases in academia.

Van de Velde and Heller take issue with the interpretation of the three-way interaction between sex of the requester, sex of the participant and condition on the likelihood of scientist sharing their science, which we found to be statistically significant in our original study^1^. They do so based on the contrasts produced by the statistical software they used. However, these contrasts are not very informative when concerning a three-way interaction. The reason being that a three-way interaction reports variation in a two-way interaction across the third level and this is not reflected in contrasts between each combination of these three factors. In our case that was the two-way interaction between sex of the requester and sex of the participant across conditions, and this was also verified by the post-hoc analyses (with corrections) that we provided in the original manuscript; i.e. both the two-way interaction being significant, and a significant difference in effect-size of this two-way interaction between the two conditions. We acknowledge that it might have been more parsimonious to test for the two-way interaction effect while controlling for condition, and are pleased to see that when doing this Van de Velde and Heller find a similar trend with respect to the two-way interaction between the sex of the requester and the sex of the participant (*P *= 0.061).

Second, Van de Velde and Heller take issue with our modeling procedure, as they argue we have violated the principle of marginality. This principle, however, has been heavily debated^2,3^, and in fact, when clear hypotheses are provided, like we did in our original study, it is even advised to deviate from this principle^4^. Importantly, however, when Van de Velde and Heller reproduce model selection according to their standards, the crucial interaction term (i.e. sex of requester with sex of participant) is maintained in the final model, and, albeit not statistically significant at *P* < 0.05, again they find a similar trend (*P* = 0.061; see the supplements to their comment).

There are multiple procedures one can follow when analyzing data. The interaction between sex of the requester and sex of the participant can also be analyzed using a dummy variable that reflects the sex combination of both (FF, FM, MF, and MM). In our original analyses (and submission) we did this and also here this dummy variable was maintained in the best fitting model, and we found that sex-combination had a significant effect on the likelihood to share a paper or data (F_3, 389_ = 4.94, *P* = 0.044), with post-hoc analyses reporting exactly the same patterns as we did in the final paper. Taken together, four different procedures of analyzing our data find a similar pattern of an interaction effect between the sex of the requester and the sex of the participant at a *P* ranging between 0.004 and 0.061, echoing our original finding.

Van de Velde and Heller argue that they do not want to defend the *P* = 0.05 threshold, yet that is exactly what they do, especially as they seem to ignore the effect size (a > 15% increase in MM responses in comparison to all other sex-combinations). Their worry about false positives is of course warranted, although their simulations of our data do not provide any out of the ordinary false positive rates. In fact, as attested by the paper of Nuzzo^5^, which Van de Velde and Heller refer to themselves, any effect that is significant at the *P* = 0.05 level has a false-positive probability of at least 29%. We acknowledge the problems with repeatability of scientific studies, particularly in the social sciences, and therefore encourage others, including Van de Velde and Heller, to replicate our study and truly test the robustness of our finding.

We do, however, in response to Van de Velde and Heller’s comment want to provide the reader with a more balanced view of the literature on gender biases in academia. Van de Velde and Heller report on two studies that did not find a pro-male bias in academia as to bolster their claim against our paper. Thereby, however, a vast body of literature e.g.^6–20^ (reviewed in^21,22^) as well as several surveys of national research councils/foundations^23,24^ is omitted.

In sum, we agree with Van de Velde and Heller that studies on gender biases in academia should be held to the same standards as any scientific claim. However, we feel that their re-analyses of our data does not harm the conclusions drawn in our original study, namely that sharing of science is more likely among male scientists. Nevertheless, we do acknowledge the importance of replication in science, and especially in such a hotly debated topic as gender biases in academia. Therefore, we encourage Van de Velde and Heller, as well as other scientists, to replicate our study and to truly test how robust and generalizable our results are.
